# Potential impact of climate change on dietary grain protein content and its bioavailability-a mini review

**DOI:** 10.3389/fnut.2024.1397219

**Published:** 2024-08-27

**Authors:** Sindhu Kashyap, Bellam H. Rajashekar Reddy, Sarita Devi, Anura V. Kurpad

**Affiliations:** ^1^Research Scholar, Manipal Academy of Higher Education, Manipal, India; ^2^Division of Nutrition, St. John’s Research Institute, St. John’s National Academy of Health Sciences (A Unit of CBCI Society for Medical Education), Bengaluru, India; ^3^Department of Physiology, St. John’s Medical College, Bengaluru, India

**Keywords:** climate change, grain protein, antinutrients, protein digestibility, dual isotope tracer technique

## Abstract

The changing global climate brings a gradual yet constant and adverse shift in crop production. Grain crop plants, particularly cereals and legumes, respond varyingly to adverse climate, including reduction in grain yield and changes to their nutrient densities. An understanding of specific changes to crop systems under differing climatic conditions can help in planning diets to meet human nutrient sufficiency. Grain protein content is also affected by adverse environmental factors. Deficits in protein yield, linked to changes in grain or seed protein and antinutrient concentrations, have been reported in major food crops when exposed to elevated carbon dioxide, high temperature, drought, and humidity. These changes, in addition to affecting the quantity of indispensable or essential amino acids (IAA), also impact their bioavailability. Therefore, it is important to assess consequences of climate change on grain protein quality. An important tool to measure grain protein quality, is measuring its digestibility at the level of the ileum and its IAA concentration, linked to a metric called the Digestible IAA Score (DIAAS). A minimally invasive technique called the dual isotope tracer technique, which measures IAA digestibility after simultaneous administration of two different intrinsically labelled protein sources, one a test protein (^2^H/^15^N) and one a reference protein (^13^C) of predetermined digestibility, has been used in evaluation of grain protein IAA digestibility, and promises more in the evaluation of changes based on climate. This review discusses climate induced changes to grain protein quality through the prism of IAA digestibility, using the dual isotope tracer technique.

## 1 Introduction

Agriculture and sustainable food production depends, in the short term on the weather and in the long term on climate (1). Recent trends in climate change have largely been attributed to emission of greenhouse gases like carbon dioxide (CO_2_), methane, nitrous oxide which has led to various important consequences such as increased atmospheric temperature, drought, and changes in rainfall pattern to name a few (2). Unseasonal environmental changes might impact seasonal crops as they require optimal conditions to achieve their vegetative and reproductive potential (1, 2). When crop plants fail to adapt to the changing environment or activate mechanisms to conserve organic matter, this results in a lower grain yield (3). Further, the mobility of reserves from leaves and roots to the grain is altered and this causes modifications in nutrient densities (4). For instance, macronutrients such as protein and micronutrients such as zinc and iron deposition in major food crops such as wheat, corn, rice, and soy decreased by approximately 3 to 9% when grown under high CO_2_ (5). Variations in nutrient densities co-occur with changes in secondary metabolite concentrations in the grain; for example, the concentration of antinutrients, such as polyphenols, can affect nutrient bioavailability from the grain (6). These shifts are also found in the vegetative parts of the crops which serve as the fodder for livestock and can potentially impact productivity in terms of meat, eggs, and milk (7, 8). These effects on food systems can be multifaceted, and therefore the evaluation of climate induced changes in food systems can help plan global food production to achieve nutrient security and sufficiency.

More specifically, the impact of climate change on protein nutrition needs evaluation as protein is critical for growth and maintenance of body structural and functional proteins encoded by the human genome (9). The grain protein quantity of cereals and legumes, grown under a predicted atmospheric CO_2_ that would occur in 2050, would decline by 4% (10). It also is important to determine the effect of climatic changes to the grain components and protein composition, and antinutrient quantity which can affect protein digestibility and hence its quality (11). The quality of a protein is dependent on the ability of its indispensable or essential amino acid (IAA) content to satisfy age-specific requirement (amino acid score) and its digestibility (digestion and absorption) (12, 13). The measurement of ‘protein digestibility’ is now recommended for individual IAA as this can vary for each IAA, either due to the difference in their interactions with the food matrix, which could include antinutrients, like anti-proteases, or due to a varied effect of food processing on different IAA (14). The digestibility of grain proteins becomes critical to measure in different food matrixes, to define protein quality and the ability of specific plant foods to meet daily protein and IAA requirements. The present review aims to understand the effect of changes in three major environmental factors such as atmospheric CO_2_, temperature and water availability, which are critical for plant growth and productivity, on grain protein yield and content, protein composition, and antinutrient concentrations. Further, the review also examines a minimally invasive method of protein digestibility measurement in humans which can be used to assess protein quality against the background of climate induced grain composition changes.

## 2 Effect of climate change on grain protein yield

Crop plants grown under elevated atmospheric CO_2_ have increased grain yield resulting from higher carbon assimilation (15, 16). Free-air-CO_2_ enrichment field experiments show that the quantum of yield increase was greater in C3 cereals, such as rice and wheat (10–12%) at CO_2_ exposure of 500 to 700 ppm compared to C4 plants such as maize (16–18). This is mainly due to increased photosynthetic efficiency of C3 plants under elevated CO_2_, while C4 plants such as maize which are already efficient photosynthetic assimilators do not respond equally to elevated atmospheric CO_2_ (15). However, increased yield is associated with ionomic imbalances and changes to protein concentration in most crop plants (5). A meta-analysis of 228 studies with elevated CO_2_ showed a reduction in the grain protein content (GPC) of major food crops such as rice, wheat, barley and soyabean (16). The decreased GPC in rice and wheat (7 to 15%) could be due to its dilution by higher quantities of carbohydrates synthesized or due to decreased leaf protein concentration which is the main source of cereal grain protein (16–18). The reduction of GPC was lower in legumes compared to non-leguminous C3 plants, except for chickpea (8–10%). Smaller but significant decreases of GPC were found in soybean (4.8%), lentil (2%), and field pea (3%) (19–22). This could be attributed to nodule-based nitrogen fixation and protein translocation to the seeds in legumes. Although GPC decreases under elevated CO_2_, the overall protein yield/hectare may not be reduced as it is compensated by increases in grain yield/hectare (23).

Decreased grain protein yield is associated with total crop yield losses. An analysis of the cereal yield, the largest contributors for global protein, over two decades (1990–2010), has shown a production plateau in major cereal producing areas across the world. This yield gain plateau could be due to maximum yield potential of these areas or due gradual climatic changes over the two decades (24). In Europe, between 1991 and 2015, cereal production decreased by 7.3% mainly due to extreme weather conditions such as heatwaves and drought (25). An increase in temperature by 1°C during cultivation of different varieties of wheat can reduce its yield by 3–10% (26). This is not limited to cereals, as the crop yield of legumes decreased by 4–31% with increases in temperature of 1–4°C (27, 28). Crop plants experience heat stress on exposure to increased temperature; and nutrient composition of grains are particularly affected if this exposure is during the reproductive and the seed filling stage as it reduces the seed filling time and impairs starch and protein synthesis (29–31). The protein content of rice and wheat on a dry weight basis increased under heat stress, but the amount of protein/grain was not altered when compared to control (32–34). However, in spring dry pea, protein/seed decreased when compared to control plants with every 1°C rise in temperature, but the magnitude of this decrease was lower compared to other dry matter components of the seed (0.032 mg of protein vs. 0.8 mg of other dry matter components/seed), such that the rise in temperature eventually resulted in an increased total grain protein on dry weight basis (35). Mixed results were observed for legumes, for instance in mung bean, lentil and chickpea, GPC reduced on an average by 7, 14, and 19% respectively, while it increased by 6.7% in soybean (36–39) (Table 1).

**Table 1:** Effect of different abiotic stresses on crop grain protein content.

Stress^a^	Crop	Treatment	Treatment stage	Grain protein content	Reference
Elevated CO_2_					
	Wheat	553 ppm	Reproductive phase	↓ 7.4%	17
	Wheat	500 ppm	Full growth cycle	↓ 14.9%	18
	Rice	500 ppm	Full growth cycle	↓ 7.0%	18
	Barley	550 ppm	Full growth cycle	↓ 11.5%	58
	Maize	550 ppm	Full growth cycle	↑ 2%	58
	Chickpea	580 ppm	Full growth cycle	↓ 8.4–10.2%	19
	Soybean	700 ppm	Seed filling	↓ 4.8%	20
	Lentil	550 ppm	Full growth cycle	↓ 2%	21
	Field pea	550 ppm	Full growth cycle	↓ 3%	22
Elevated temperature					
	Rice	↑ 5°C average air temperature	Grain filling	↑ 21%	32
	Wheat	From 24°C/16°C to 35°C/25°C	25 days post anthesis	↑ 6.6%	33
	Spring dry pea	↑ 5°C average air temperature	Grain filling	↑ 6.2%	35
	Mung bean	↑ 4°C average air temperature	Full growth cycle	↓ 4.1 to 9.3%	36
	Chickpea	From 25/15°C to 32/20°C	Grain filling	↓ 19%	37
	Lentil	From 11.4–30.6°C to 22.4 to 43°C	Reproductive phase	↓ 14%	38
	Soybean	↑ 6°C average air temperature	Grain filling	↑ 6.7%	39
	Sorghum	From 22.5°C to 30.6°C	Full growth cycle	↓ 9%	44
Drought					
	Wheat	Half optimal irrigation	Full growth cycle	↑ 15–18%	84
	Mung bean	No irrigation	Reproductive phase	↑ 10%	41
	Chickpea	Gradual evaporative water deficit	Full growth cycle	↓ 5%	41
	Common bean	No irrigation	Reproductive phase	↑ 6–10%	42
	Faba bean	No irrigation	Full growth cycle	↓ 12%	43
	Sorghum	No irrigation	Full growth cycle	↑ 8%	44
Combined stress					
Heat and CO_2_	Rice	↑ 5°C and 700 ppm	Reproductive phase	↓ 4–6%	45
Heat, CO_2_, and ozone	Wheat	↑ 5°C, 700 ppm and 80–100 ppb	Reproductive phase	↑ 4.6%	46
Heat and drought	Lentil	↑ 19°C and without irrigation	Reproductive phase	↓ 57.2%	38

^*^CO_2_, carbon dioxide; ppm, parts per million; ppb, parts per billion.

a Values are a mean if multiple varieties are evaluated by a study.

Water deficit (drought) which often accompanies increases in temperatures also has varying effects on grain protein yield. A meta-analysis of 48 studies on effect of drought on wheat showed a decrease in grain yield and grain protein yield/hectare by 57.32 and 46.04%, respectively, but GPC increased by 9.38% (40, 41). This effect is similar to that observed under high temperature. Decreased protein yield/hectare was also reported in chickpea and mung bean under drought where protein yield reduced by 41 and 88%, respectively, (42). While GPC decreased in chickpea by 5% and faba bean by 12%, it increased in mung bean, and a few common bean varieties by 10% and 6–10%, respectively, (42–44). In sorghum, while heat stress on an average decreased GPC in 24 different cultivars by 9%, drought increased it by 8% (45). Although it is important to understand the effect of individual climatic changes, cultivated lands experience multiple stresses together; therefore, their simultaneous interaction on the GPC needs to be considered. For instance, rice cultivars grown under elevated CO_2_ and heat had 4–6% lower GPC when compared to elevated CO_2_ alone (46). In wheat, combined stress of ozone (80–100 ppb), elevated CO_2_ (700 ppm), and higher temperature (5°C) increased GPC by 4.6% (47) (Table 1). Combined heat and drought stress reduced GPC by 57% in lentil; however, this reduction in GPC was 14% when grown under heat stress alone (38). Overall, grain protein yield is affected by reduction of crop yield or reduction of GPC from exposure to various weather/climatic conditions. Selection of cultivars which are tolerant to different abiotic stresses with minimum yield and grain protein penalty can help in future climatic conditions. However, these varieties might also have increased quantities of antinutrients such as polyphenols and phytic acids which accumulate in grain in response to abiotic stresses (48, 49).

## 3 Effect of climate change on grain protein composition, amino acid and antinutrient concentration

The shifting climate not only changes the proximate nutritional make-up of the grain, but also leads to changes in the protein fractions deposited in the grain. The main storage proteins of cereals and legumes are albumin, globulin, prolamin and glutelins with crop specific variations in their type, proportions and subfractions (50). Climate change induced changes can co-occur in all the storage protein fractions, however the extent to which each protein fraction is affected defines the grain protein digestibility. This is because each protein fraction is digested with different efficiencies in different crops. In rice bran protein when assessed *in vitro*, glutelin had highest digestibility followed by albumin and globulin, and prolamin (51). Whereas in barley, hordeins (prolamins) had the highest digestibility followed by albumin and globulin, and glutelin (52).

A change in gliadin to glutenin ratio (reduction by 4.8%) was observed when wheat was grown under elevated CO_2_ conditions (53). The reduced gliadin to glutenin ratios could decrease the protein digestibility of wheat as the glutenin fraction is 6% less digestible than the gliadin fraction (54). On the contrary, the protein digestibility of maize grown under elevated CO_2_ was 9% higher in pigs, although the changes in the protein fractions were not reported in this study (55). Elevated CO_2_ decreased albumin (34%), prolamin (21%), glutelin (17%) and globulin (16%) concentrations in rice (56). A concurrent decrease in of all IAA concentrations in the range of 4.8 to 9.0% in wheat and 1.6 to 5.0% in rice was observed when grown under elevated CO_2_ (18). Heat stress that is experienced after anthesis in wheat also resulted in changes of gluten protein composition by decreasing the ratio of gliadin to glutenin (5.5%) (57). In rice, exposure to high temperature caused a reduction in prolamin by 12% and increase in glutelin by 31% (58). Changes in protein fractions were also observed in other crops including maize, barley, mung bean, lentils, and chickpea, at elevated CO_2_ and under heat stress (Table 2) (36, 37, 59, 60). It is important to note that the changes in protein fractions of the grains are dependent on various factors including the length and time of stress induction, variety of the crop, the type of stress induced and combination of environmental stresses that the crop experiences. In total, the alterations in AA concentration and protein fraction of grains have potential to influence their protein digestibility and quality.

**Table 2:** Effect of different abiotic stresses on grain protein composition, amino acid and antinutrient concentration.

Stress^a^	Crop	Treatment	Treatment stage	Protein composition, IAA and antinutrient concentration	Reference
Elevated CO_2_					
	Wheat	550 ppm	Full growth cycle	↓ 4.8%: gliadin to glutenin ratio	52
		500 ppm	Full growth cycle	↓ 7%: threonine, ↓ 7.5%: valine, ↓ 7.1%: methionine, ↓ 9%: isoleucine, ↓ 8.1%: leucine, ↓8.2%: phenylalanine ↓ 4.8%: lysine	18
		553 ppm	Reproductive phase	↑ 5%: fructose, ↑ 4%: fructan	17
	Rice	↑ 200 ppm	Full growth cycle	↓ 34%: albumin, ↓ 21%: prolamine, ↓ 17: glutelin, ↓ 16%: globulin	55
		500 ppm	Full growth cycle	↓ 1.6%: threonine, ↓ 4.5%: valine, ↓ 5.0%: methionine ↓ 1.9%: isoleucine ↓ 1.7%: leucine, ↓ 1.5%: phenylalanine, ↓ 2.6%: lysine	18
	Barley	550 ppm	Full growth cycle	↓ 34%: albumin, ↓ 2.3%: globulin, ↑ 12%: glutenin, ↑ 10%: horedins	58
	Maize	550 ppm	Full growth cycle	↓ 32%: albumin, ↓ 62%: globulin, ↑ 37%: glutenin, ↑ 14%: zien	58
	Faba bean	700 ppm	Full growth cycle	↑ 53%: total phenolic	65
Elevated temperature					
	Wheat	24/17°C to 37/28°C	At anthesis	↓ 5.5%: gliadin to glutenin ratio, ↓ 40%: albumin and globulin	46
	Rice	↑ 1.6°–3.1°C	Grain filling	↓ 12%: Prolamin, ↑ 31%: Glutelin	57
	Chickpea	From 25/15°C to 32/20°C	Grain filling	↓ 37.6%: globulins, ↓ 14.6%: glutenins, ↓ 29%: prolamins, ↓ 27.8%: albumins	37
				↑ 43% glucose and ↑ 49.5%: fructose	37
	Lentil	From 28/23°C to 33/28°C	Post anthesis	↓ 21%: albumin, ↓ 14%: globulin, ↓ 22%: glutelins, ↓ 28.2%: prolamins	59
				↓ 19.2: methionine + cystiene, ↓ 21.3%: phenylalanine and tyrosine ↓ 14.7: threonine, ↓ 8.4%: tryptophan	59
	Rice	↑ 6°C	At anthesis	↑ 13.7%: phytic acid	60
	Lentil	↑ 10°C	Reproductive phase	↑ 11%: phytic acid	61
	Sorghum	From 32°C/21°C to 38°C/21°C	Full growth cycle	↑ 29.2%: phytic acid	62
	Wheat	↑ 5°C and ↑ 10°C	Full growth cycle	↑ 15.6% and ↑ 30.6%: total phenolic	64
	Wheat	From 22/12°C to 32/22°C	Reproductive phase	↑ 11%: arabinoxylan	66
		Reduction in soil moisture by 60%	Stem elongation stage	↑ 10%: arabinoxylan	

^*^CO_2_, carbon dioxide; ppm, parts per million.

a Values are a mean if multiple varieties are evaluated by a study.

Environmental changes affect the quantity of antinutrients such as phytic acid (PA) and phenolics in grains. For example, PA concentrations increased by an average of 13.7% in 22 rice genotypes when exposed to heat stress, and the extent of this increase varied among different cultivars (61). Increases in PA concentration were found in lentil and sorghum while there was no change in wheat (62, 63). Elevated CO_2_ decreased PA content in wheat, while increases were observed in rice (5, 64) (Table 2). High temperature increased total phenolic content (free and bound) in wheat genotypes (4–33% for every 5°C increment) while elevated CO_2_ increased total phenolics in faba beans by 50% (65, 66). Both PA and phenolics can decrease protein digestibility by 3–31%, although the extent of this decrease varies on the concentration of PA and the type of polyphenols (11). A higher quantity of non-structural carbohydrate is observed in a few forms of abiotic stress. Under elevated CO_2_, wheat grains had significantly higher concentrations of fructose (5%) and fructan (4%). The concentrations of other carbohydrates such as sucrose, raffinose and maltose also increased, though this the change was not significant (17) (Table 2). The presence of these sugars, in excess, indirectly influences protein quality as they can be involved in Maillard reactions with grain protein during processing for consumption and form products which are not utilized functionally (11). Non-structural polysaccharides which impact protein digestibility such as arabinoxylan has been shown to increase in spring wheat by 11 and 10% under heat and drought stress, respectively, (67). Together these changes can result in a decrease of protein quality. While limited *in vitro* studies examine the digestibility under different abiotic stresses, there are no studies which examine the effect of climate on grain protein digestibility in humans. This is important as in addition to the above-mentioned factors, food matrices in which protein is habitually consumed can further affect bioavailability.

## 4 Protein digestibility measurement and the digestible indispensable amino acid score

The changing landscape of dietary protein quantity in the background of differing grain components make it important to measure protein digestibility in widely cultivated and emerging abiotic stress resistant crop cultivars, and alternative protein sources. It is also important for understanding the effectiveness of food processing techniques on improving protein digestibility. A protein quality metric which takes into account the amino acid score as well as its digestibility (digestion and absorption till the terminal ileum), called the digestible indispensable amino acid score (DIAAS), is currently recommended for protein quality assessment (12, 13), and briefly described below.

Protein digestibility was earlier measured by oro-fecal intestinal balance, as the difference in the protein content between intake and fecal excretion, expressed as a proportion of the intake (14). The colonic microbial protein transactions that trap body nitrogen in the colon (urea for example) can confound these measurements (14). Since digestion and absorption of dietary protein is mainly considered to occur in the small intestine, measurement of ileal amino acid digestibility (oro-ileal balance) is recommended, corrected for the contribution from endogenous protein secretions (14). Ileal digestibility measurements are invasive as the ileum is not easily accessible and requires measurement of the endogenous intestinal protein secretions as well. It is measured by naso-ileal intubation technique or fistulation of the terminal ileum to collect ileal effluents required for quantification of the amount of ingested protein that disappeared after digestion and absorption until the terminal ileum (14). As stated above, substantial amounts of endogenous protein, which are secreted into gastrointestinal tract, mix with the dietary protein leading to an underestimation of oro-ileal digestibility (14, 68). Therefore, an additional measure of endogenous protein losses on a separate day becomes necessary (14). When corrected for endogenous protein losses, the ileal IAA digestibility is termed as “true ileal IAA digestibility.” Using stable isotopically labelled test dietary protein which distinguishes it from the endogenous protein helps avoid this additional measurement (14). Due to the invasiveness of the ileal-balance method, it cannot be used widely to determine ileal protein digestibility.

A relatively recent, minimally invasive technique called the dual isotope tracer technique is promising for measurement of true IAA digestibility in different populations and age groups (69). In this technique, two intrinsically stable isotopically labelled protein, one a test protein (^2^H/^15^N) and another a differently labelled reference protein (^13^C) of known digestibility, are simultaneously administered in a plateau feeding protocol. The ratio of postprandial plasma enrichment of ^2^H/^15^N IAA from the test protein and ^13^C-IAA from the reference protein at plateau corrected for amounts ingested and the digestibility of the reference protein provides a measure of test protein digestibility (69). The equation used for the calculation of true IAA digestibility by dual isotope tracer technique is given below:


$$\begin{array}{l} {\mathrm{Dig}}_{\mathrm{test}}=\left[{\mathrm{Plasma}}^{2}H-IAA\;\left(\mathrm{APE}\right)/{\mathrm{Meal}}^{2}H-IAA\;\left(\mathrm{APE}\right)\right]/\\ {\left[{\mathrm{Plasma}}^{13}C-IAA\;\left(\mathrm{APE}\right)/{\mathrm{Meal}}^{13}C-IAA\;\left(\mathrm{APE}\right)\right]}^{*}\;\\ 100^{*}\;{\mathrm{Dig}}_{ref}/100. \end{array}$$

Where, Dig_test_ is digestibility of test protein, Dig_ref_ digestibility of reference protein, and APE is atom percent excess.

The dual isotope tracer technique makes two important assumptions, first, that the absorption kinetics of labelled IAA from both the test and reference protein are similar. Second, differently labelled IAA’s undergo similar splanchnic extraction and metabolism (14). Both of these assumptions are reasonable as an isotopic effect for these processes is not conclusively known. Several aspects of the measurement, to be considered while using this technique, such as intrinsic labelling of the test protein, selection of reference protein and feeding protocol have been discussed before (70).

To obtain intrinsically labelled plant protein, different methods are available, through foliar or soil applications of ^15^N labelled ammonium/potassium salts or by administration of heavy water (^2^H_2_O) to the soil, or through hydroponics during the reproductive and seed development phase of the plant (69, 71–73). ^15^N-salts label the amino groups of all IAA, while deuterium atoms from ^2^H_2_O are fixed at different position into IAA during their synthesis (73). The extent of labelling of the IAA depends on the quantity of the precursors administered and the length of application. The incorporation of ^15^N into IAA is more efficient than ^2^H because ^15^N is incorporated into fewer molecules such as protein, and nucleic acids whereas ^2^H is incorporated into all molecules. It is important to consider losses of label which can occur during metabolic reactions, for instance, ^15^N from α-amino groups of labelled IAA are replaced by ^14^N during the reverse reaction of transamination and the α-^2^H of AA is replaced with H from body water (74, 75). Transamination correction factors can be derived in separate studies to account for these losses (69, 76).

The reference protein used could either be a ^13^C-labelled bound protein of known digestibility or free ^13^C-AA mixture (69, 77). Commercially available U-^13^C spirulina has been previously used as the reference protein in dual isotope tracer technique; however, the interindividual variability of spirulina IAA digestibility was found to be high (1–12%) (69). Animal source protein (egg, milk, whey, or casein) with high digestibility and lower inter-individual variability can also be used as reference protein. The other option is to use ^13^C-AA mixture which is considered to have 100% digestibility. A protein comparator as a reference is preferred as peptides have an absorption advantage over free AA (78).

The dual isotope tracer technique has not yet been validated with ileal-balance methods in an appropriately designed protocol. However, the true ileal IAA digestibility of animal source foods measured by dual isotope tracer technique was similar to that measured by other ileal balance methods (79–82). Previous studies have shown that the mean true ileal IAA digestibility of *desi* chickpea and *kabuli* chickpea was estimated to be 56 and 74.6%, respectively, and extrusion increased this by 89% (69, 77, 83). Climate change induced increases in grain anti-nutrients, such as polyphenols and phytate, (65, 66) can potentially decrease grain protein digestibility through covalent or non-covalent interactions with either the grain protein or the gastrointestinal tract proteases which hydrolyze them. This effect on digestibility can be shown through traditional processing techniques such dehulling, which has been shown to increase the mean true IAA digestibility of whole mung bean by 7.7% (77). Dehulling removes antinutrients such as tannins and polyphenols present in the seed coat which can reduce protein digestibility. Further, the true mean IAA digestibility of egg protein decreased by 17% when it was co-ingested with black tea (84), with a polyphenol content of 4.6 mg/mL.

The main advantage of the dual isotope tracer technique over the traditional oro-ileal balance methods of digestibility measurement is that it is minimally invasive. The plateau feeding protocol employed in the dual isotope tracer technique minimizes the number of blood samples collected and hence it can be used across age groups. The use of intrinsically labelled protein prevents confounding by endogenous protein secretions and allows measurement of true digestibility of all IAA of a grain protein on a single study day. The technique is also sensitive to changes in true IAA digestibility of different crop varieties, varying food matrices, and food processing techniques. Therefore, it has the potential to be used to assess the effect of climate change on true IAA digestibility of different dietary grain protein sources across geographical locations in different populations.

## 5 Conclusion and future research directions

Climate change has the potential to reduce protein quality particularly in crop grains, by reduction in protein yield, protein content, IAA content, and decreased digestibility due to protein compositional changes and varied antinutritional factors. Systematic field-based analysis of effect of combined abiotic stresses on protein content and protein quality is required to plan a sustainable approach for improving protein nutrition. Mitigation strategies including selection of cultivars which are tolerant to environmental stresses, introgression of multiple abiotic stress tolerant traits in new cultivar by crop breeding, and alternative sources of protein needs to be evaluated. In addition to traditional crop breeding techniques, newer approaches such as quantitative trait loci mapping and marker assisted section, and CRISPR-Cas9 genome editing can be used to introduce multi-stress tolerances in important food crops. Selection of alternative crops to suit the climate of a particular production area to diversity cropping systems can help in sustaining or increasing nutrient production/hectare. Combination of different food processing techniques or development of processing techniques to reduce the effect of anti-nutrients can further improve protein quality. The dual isotope tracer technique, while expensive, is minimally invasive and can be used to evaluate the effect of changing climate on the protein digestibility of crops in humans and to determine the effectiveness of mitigation strategies in improving protein digestibility. It is also important to determine the fate of higher quantities of undigested crop protein which can enter the colon in background of climate change with controlled intervention studies, as several beneficial and adverse effects on health outcomes have been associated with protein fermentation products in the human colon.
